# Dose-dependent effect of GFI1 expression in the reconstitution and the differentiation capacity of HSCs

**DOI:** 10.3389/fcell.2023.866847

**Published:** 2023-04-05

**Authors:** Xiaoqing Xie, Pradeep Kumar Patnana, Daria Frank, Judith Schütte, Yahya Al-Matary, Axel Künstner, Hauke Busch, Helal Ahmed, Longlong Liu, Daniel R. Engel, Ulrich Dührsen, Frank Rosenbauer, Nikolas Von Bubnoff, Georg Lenz, Cyrus Khandanpour

**Affiliations:** ^1^ Department of Medicine A, Hematology, Oncology, and Pneumology, University Hospital Münster, Münster, Germany; ^2^ Department of Hematology-Oncology, Chongqing University Cancer Hospital, Chongqing, China; ^3^ Department of Hematology and Stem Cell Transplantation, University Hospital Essen, Essen, Germany; ^4^ Department of Hematology and Oncology, University Hospital Schleswig-Holstein, University of Lübeck, Lübeck, Germany; ^5^ Department of Dermatology, University Hospital Essen, Essen, Germany; ^6^ Institute of Experimental Dermatology, University of Lübeck, Lübeck, Germany; ^7^ Department of Immunodynamics, Institute for Experimental Immunology and Imaging, University Hospital Essen, Essen, Germany; ^8^ Institute for Molecular Tumor Biology, University Hospital Münster, Münster, Germany

**Keywords:** Gfi1, HSC, dose-dependent, engraftment, differentiation

## Abstract

GFI1 is a transcriptional repressor and plays a pivotal role in regulating the differentiation of hematopoietic stem cells (HSCs) towards myeloid and lymphoid cells. Serial transplantation of *Gfi1* deficient HSCs repopulated whole hematopoietic system but in a competitive setting involving wild-type HSCs, they lose this ability. The underlying mechanisms to this end are poorly understood. To better understand this, we used different mouse strains that express either loss of both *Gfi1* alleles (*Gfi1*-KO), with reduced expression of *GFI1* (*GFI1*-KD) or wild-type *Gfi1/GFI1* (*Gfi1*-/*GFI1*-WT; corresponding to the mouse and human alleles). We observed that loss of *Gfi1* or reduced expression of *GFI1* led to a two to four fold lower number of HSCs (defined as Lin^−^Sca1^+^c-Kit^+^CD150^+^CD48^−^) compared to *GFI1*-WT mice. To study the functional influence of different levels of GFI1 expression on HSCs function, HSCs from *Gfi1*-WT (expressing CD45.1 + surface antigens) and HSCs from *GFI1*-KD or -KO (expressing CD45.2 + surface antigens) mice were sorted and co-transplanted into lethally irradiated host mice. Every 4 weeks, CD45.1+ and CD45.2 + on different lineage mature cells were analyzed by flow cytometry. At least 16 weeks later, mice were sacrificed, and the percentage of HSCs and progenitors including GMPs, CMPs and MEPs in the total bone marrow cells was calculated as well as their CD45.1 and CD45.2 expression. In the case of co-transplantation of *GFI1*-KD with *Gfi1*-WT HSCs, the majority of HSCs (81% ± 6%) as well as the majority of mature cells (88% ± 10%) originated from CD45.2 + *GFI1*-KD HSCs. In the case of co-transplantation of *Gfi1*-KO HSCs with *Gfi1*-WT HSCs, the majority of HSCs originated from CD45.2+ and therefore from *Gfi1*-KO (61% ± 20%); however, only a small fraction of progenitors and mature cells originated from *Gfi1*-KO HSCs (<1%). We therefore in summary propose that GFI1 has a dose-dependent role in the self-renewal and differentiation of HSCs.

## 1 Introduction

Gfi1 (Growth factor independence 1) is a transcriptional repressor with an important role in the function and differentiation of hematopoietic stem cells (HSCs) in the direction of myeloid and lymphoid lineage (Phelan et al., 2010; van der Meer et al., 2010). It regulates the maturation of myeloid cells and is required for maintaining different signalling pathways such as Notch signalling (Horman et al., 2009; Velu et al., 2009; Phelan et al., 2013). Analysis of *Gfi1* expression in *Gfi1*:GFP heterozygous knock-in mice revealed that *Gfi1* is primarily expressed in HSCs, granulocyte-macrophage progenitors (GMPs) and common lymphoid progenitors (CLPs), while it is absent in common myeloid progenitors (CMPs) and megakaryocyte-erythroid progenitors (MEPs) (Zeng et al., 2004). *Gfi1*-deficient mice are characterized by an accumulation of monocytic cells and an absence of granulocytes, leading to severe neutropenia and monocytosis (Karsunky et al., 2002a; Hock et al., 2003). Recently it has been shown that reduced expression of *GFI1* in blast cells of myeloproliferative neoplasm (MPN), chronic myeloid leukemia (CML) and acute myeloid leukemia (AML) patients are associated with an inferior prognosis and event-free survival (Hock et al., 2004; Khandanpour et al., 2012; Fraszczak et al., 2019). We have also shown that reduced expression of *Gfi1* accelerated leukemia development in the murine model of AML (Kok et al., 2013; Hönes et al., 2016; Vadnais et al., 2018). These studies thus also underscore the contribution of Gfi*1* in the expression of oncogenes, apoptotic pathways and metabolic functions in a dose-dependent manner. However, it has also been postulated that the loss of Gfi1 negatively influences the repopulation capacity of HSCs (Hock et al., 2004; Zeng et al., 2004).

We thus explored whether the low level and loss of *Gfi1* indeed negatively affected the stem cell capacity of HSCs and how the seemingly contradictory findings between reduced self-renewal capacity and the dose-dependent role of *Gfi1* function in myeloid pathogenesis could be reconciled. To reach this goal we made use of different mouse strains: *Gfi1*-knockout (KO) mice, (with a complete loss of Gfi1) and *GFI1*-knockdown (KD) mice, a mouse strain in which the human *GFI1* was cloned into the murine *Gfi1* gene locus together with a Neo cassette in the opposite direction of transcription, leading to lower *GFI1* expression (10%–15% of wild-type levels) (Hönes et al., 2016). We now show that *GFI1*-KD and *Gfi1*-KO mice feature an elevated number of hematopoietic progenitors (LSK cells, Lin^−^Sca1^+^c-Kit^+^), but lower HSC (Lin^−^Sca1^+^c-Kit^+^CD150^+^CD48^−^) numbers. In contrast to *Gfi1*-KO HSCs, *GFI1*-KD HSCs can compete with *Gfi1*-WT HSCs in a competitive transplantation setting and contribute to multi-lineage differentiation. On the other hand, *Gfi1*-KO HSCs can only engraft but are not able to expand and lose their ability to contribute to multi-lineage differentiation.

## 2 Methods

### 2.1 Mice models

*GFI1*-36S (*GFI1*-WT), *Gfi1*-knock out (KO) and *GFI1*-knock down (KD) mice have previously been described (Karsunky et al., 2002a; Hönes et al., 2016; Hönes et al., 2017). *GFI1-*WT mice and the *GFI1-*KD mice express the human *GFI1* instead of the murine *Gfi1*. B6.SJL-Ptprca Pepcb/BoyCrl (CD45.1) mice were purchased from Charles River (Charles River Europa, Italy). Mice were housed in specific pathogen-free conditions at the animal facility of University Hospital Muenster, Germany and the animal facility of University Hospital Essen, Germany. All experiments were approved by the local authorities (LANUV) of North Rhine-Westphalia (AZ84-02.04.2015.A022 and AZ81-02.04.2021.A150).

### 2.2 FACS-antibodies

The FACS antibodies used in the current study are listed in Supplementary Table S1.

### 2.3 Hematopoietic stem cells (HSCs) isolation

Bone marrow (BM) cells were isolated from tibiae, femora and humeri bones and red blood cells were lysed using 1 mL 1x BD Pharm Lyse™ (555,899, BD Biosciences) for 7 min at room temperature. Lineage-negative cells were isolated using Lineage Cell Depletion Kit (130-090-858, Miltenyi Biotec GmbH) and magnetic separation using MACS Separators and LS Columns (130-042-401, Miltenyi Biotec GmbH) following manufacturer’s instructions. Lineage-negative cells were then stained with the antibodies as shown in Supplementary Table S1 and the HSCs, which were defined as Lin^−^Sca1^+^c-Kit^+^CD150^+^CD48^−^ cell population (Supplementary Figure S1), were isolated by a flow cytometer sorting machine (FACS Aria III cell sorter, BD).

### 2.4 Colony-forming unit (CFU) assay

To perform the CFU assay, the 100 HSCs from each mouse were FACS sorted into mouse methylcellulose media MethoCult™ GF M3434-stem cell technologies) in a 24-well plate with. 5-FU was added to the methylcellulose media in the treated samples. The plate was incubated for 2 weeks without disturbing at 37°C and the number of colonies was counted after 2 weeks.

### 2.5 Competitive bone marrow transplantation assay

For co-transplantation experiments, 8–12 weeks old CD45.1 recipient mice (*Gfi1*-WT) were used and were lethally irradiated (7Gy + 3Gy) 1 day before the transplantation. Mice were irradiated using the X-Rad320, the MultiRad 225 or the CP-160 irradiation devices from Precision X-ray. To measure the competitive efficacy 200 HSCs from either *GFI1*-KD or *Gfi1*-KO mice were transplanted (i.v. into the tail vein) together with 200 HSCs from CD45.1 mice and 500.000 total bone marrow cells from the HSC donor mice (1:1 ratio) into lethally irradiated CD45.1 recipient mice.

### 2.6 Flow cytometry

#### 2.6.1 Analysis of peripheral blood

The peripheral blood (PB) of the transplanted mice was analysed every 4 weeks and directly after the mice were euthanized at week 16 after transplantation. Around 50–100 µL blood was collected from the mice’s tail vein and red blood cells were removed by using BD Pharm Lyse™ (555,899, BD Biosciences) as described above. To differentiate the various blood cell types by FACS analysis, the antibodies used were shown in Supplementary Table S1. Cell populations were measured using an Attune NxT flow cytometry from Invitrogen and the data were analyzed by FlowJo (version 10.7.2).

#### 2.6.2 Analysis of bone marrow cells

16 weeks after the transplantation, the mice were euthanized and bone marrow cells were isolated and the red blood cells were lysed as described above. Bone marrow cells were then analysed by FACS for the mature cell types, progenitor cells [Granulocyte-monocyte progenitors (GMPs), common myeloid progenitors (CMPs) and megakaryocyte-erythroid progenitors (MEPs)] and HSCs. The antibodies used for FACS staining are listed in Supplementary Table S1 and the gating for LSKs, HSCs, CMPs, GMPs and MEPs was performed as described previously (Khandanpour et al., 2012) and is shown in Supplementary Figure S1. All the cell populations were measured using an Attune NxT flow cytometry from Invitrogen and the data were analysed by FlowJo (version 10.7.2).

### 2.7 RNA-seq data analysis

#### 2.7.1 RNA-seq data quantification

The RNA-seq data (GEO accession number—GSE225653) could be accessed by the token number ‘wjsvmoimdpclzqt’. The data (single-end fastq files) quantification was performed using SALMON (v1.9.0) against the *Mus musculus* cDNA database (GRCm38, Ensembl release 102; *kmer* size set to 31). The algorithm was run using 30 bootstrap intervals, GC bias, and sequence-specific bias correction. Additionally, positional bias correction was performed, and the library type was inferred automatically by SALMON.

#### 2.7.2 Differential expression analysis and pathway enrichment

Ensembl transcript IDs were mapped to gene symbols using annotables (v0.1.91). Effect sizes (*log*_*2*_ fold changes) of gene expression differences were estimated using Wald-test in sleuth (v0.30.0) and *p*-values were retrieved from the likelihood-ratio test. Pathway enrichment analysis against combined REACTOME and HALLMARK gene sets (*msigdf* R package v7.4) on effect sizes was performed using a rank-MANOVA-based approach as implemented in *mitch* (v1.8.0; priority on significance); gene sets with an adjusted *p*-value below 0.05 and an absolute enrichment above 0.1 were considered as significant. Analysis was performed using R (v4.2.1) and tidyverse (v1.3.2) package was used for data handling. Plots were generated with the packages ggplot2 (v3.3.6). Differentially expressed genes were identified using *p*-values (*p* < 0.01).

### 2.8 Statistical analysis

Statistical analyses were performed using GraphPad Prism 9. Significance was calculated using a paired two-sided *t*-test with a normal distribution. ANOVA was used for multiple comparisions. *p* values ≤0.05 were considered significant. All methods were performed under the relevant guidelines and regulations.

## 3 Results

**FIGURE 1:**
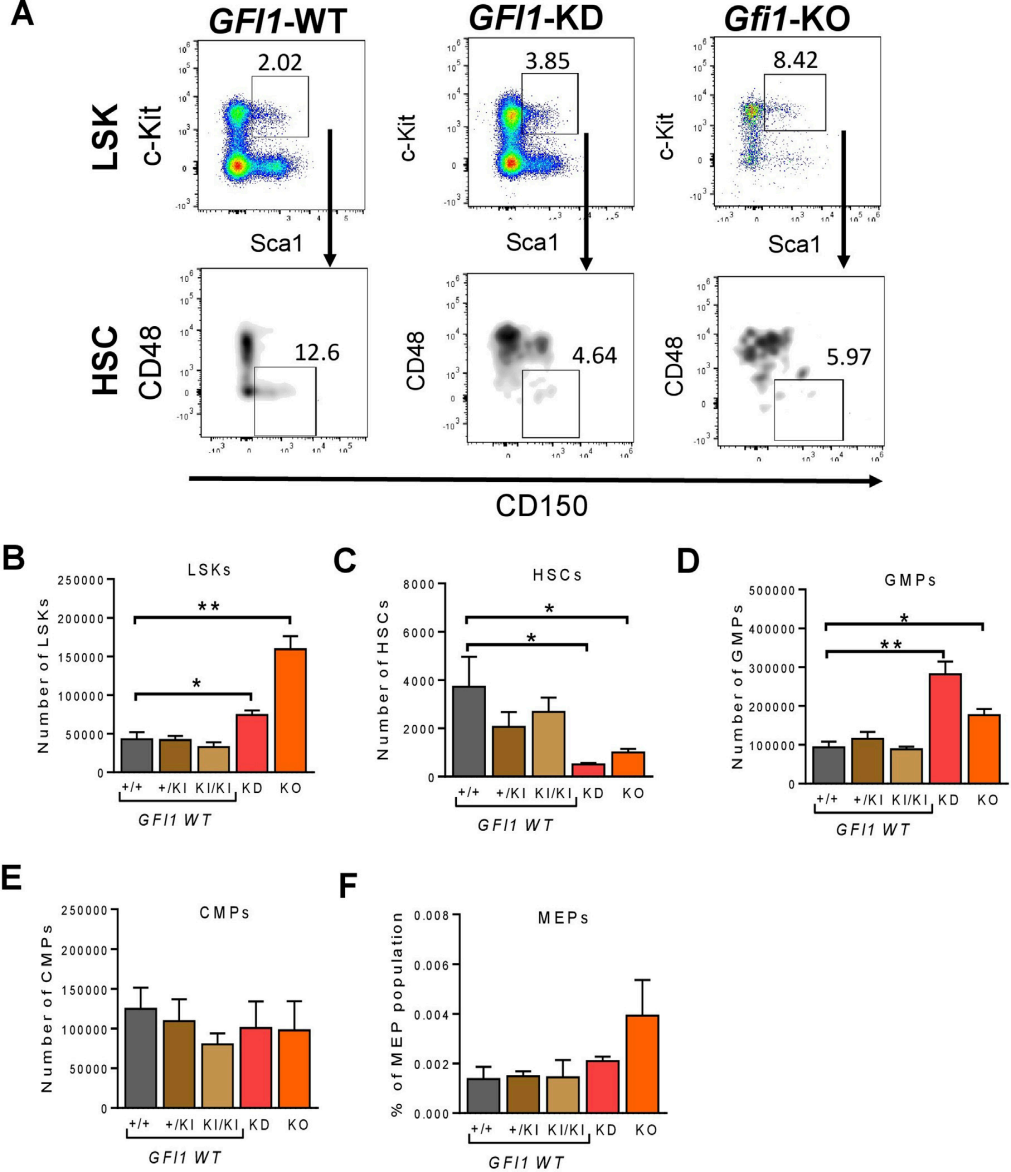
Gfi1 is required for HSC generation. **(A)**. Gating strategy for the identification of LSKs and HSCs. Murine BM cells were isolated, stained and gated for surface marker’s expression (lineage marker, c-Kit, Sca1, CD48 and CD150) by flow cytometry. Representative flow cytometry data (*GFI1*-WT, *GFI1*-KD and *Gfi1*-KO mice) are shown. **(B)**. Reduced levels of *GFI1* (*GFI1*-KD) and loss of *Gfi1* (*Gfi1*-KO) led to an increase in LSK (Lin^−^Sca1^+^c-Kit^+^) cell number compared to *GFI1* WT (+/+, +/KI, KI/KI). Depicted is the number of LSK cells in the total BM cells. **(C)**. Knockdown or loss of *GFI1/Gfi1* resulted in a significant decrease in the number of HSCs (Lin^−^Sca1^+^c-Kit^+^CD150^+^CD48^−^). **(D–F)**. The bar plots show the number of hematopoietic progenitor cells (GMPs, CMPs and MEPs) in *GFI1* WT (+/+, +/KI, KI/KI), *GFI1*-KD and *GFI1*-KO mice (n ≥ 3; Avg ± SEM; *p ≤ 0.048; **p = 0.0091; ***p = 0.0004).

We used previously described *GFI1*-KD mice (express 15%–20% of wildtype *GFI1*) (Hönes et al., 2016) and *Gfi1*-KO mice with a complete loss of Gfi1 expression (Karsunky et al., 2002a). *GFI1*-WT (KI/KI) mouse strain expresses the human *GFI1* at the murine locus and the *Gfi1-*WT (+/+) mouse strain expresses murine *Gfi1* were used as controls. We previously showed that *GFI1*-WT (KI/KI) mice containing the human *GFI1* cDNA are functionally equivalent to murine *Gfi1-*WT (+/+) mice (Botezatu et al., 2016; Hönes et al., 2016; Hönes et al., 2017). Here we initially measured the percentage of LSKs, HSCs and progenitors in *Gfi1*-WT (+/+), heterozygous knock-in (*GFI1*-+/KI) and homozygous knock-in (*GFI1*-KI/KI) and *Gfi1*-KO mice from the total bone marrow (BM) cells. BM-derived LSKs (Lin^−^Sca1^+^c-Kit^+^) and HSCs (Lin^−^Sca1^+^c-Kit^+^CD150^+^CD48^−^) were gated as shown in Figure 1A; Supplementary Figure S1. Our data indicated that *Gfi1*-WT (+/+), *GFI1* +/KI and *GFI1*-KI/KI mice do not differ in the number (Figures 1B–F) and percentage (Supplementary Figures S2A–G) of LSKs, HSCs and progenitor cells. Thus, *Gfi1*-WT (+/+) mice were used as a control in further experiments.

We next examined the frequency of hematopoietic progenitor cells in *GFI1*-WT (+/+, +/KI and KI/KI), *GFI1*-KD and *Gfi1*-KO mice. The total cell number and the percentage of LSK cells were significantly increased in *GFI1*-KD and *Gfi1*-KO mice compared to *GFI1-*WT mice (Figure 1B; Supplementary Figure S2A). In contrast, the number and percentage of HSCs were significantly reduced in *GFI1*-KD and *Gfi1*-KO mice compared *to GFI1-WT* mice (Figure 1C; Supplementary Figure S2B). We then assessed the percentage of LSKs and HSCs in old and young mice to examine if this effect was irrespective of the age of the mice. We did not observe a significant increase in LSKs but a significant reduction of HSCs in *GFI1*-KD and *Gfi1*-KO mice in both old and young groups (Supplementary Figures S2C, D). Our data thus recapitulate previous findings of our and other groups regarding an expansion of LSKs upon loss of *GFI1* (Hock et al., 2003; Khandanpour et al., 2011). Our data further expands our knowledge concerning a dose-dependent effect of *GFI1* expression on HSCs resulting in a significant decrease in HSCs number. In addition to LSKs and HSCs, we also quantified progenitor cells. Reduced expression of *GFI1* (*GFI1*-KD) or loss of *GFI1* (*Gfi1*-KO) significantly increased the number and percentage of GMPs (Figure 1D; Supplementary Figure S2E), but no changes in the number and percentage of CMPs or MEPs were observed (Figures 1E, F, Supplementary Figures S2F, G).

**FIGURE 2:**
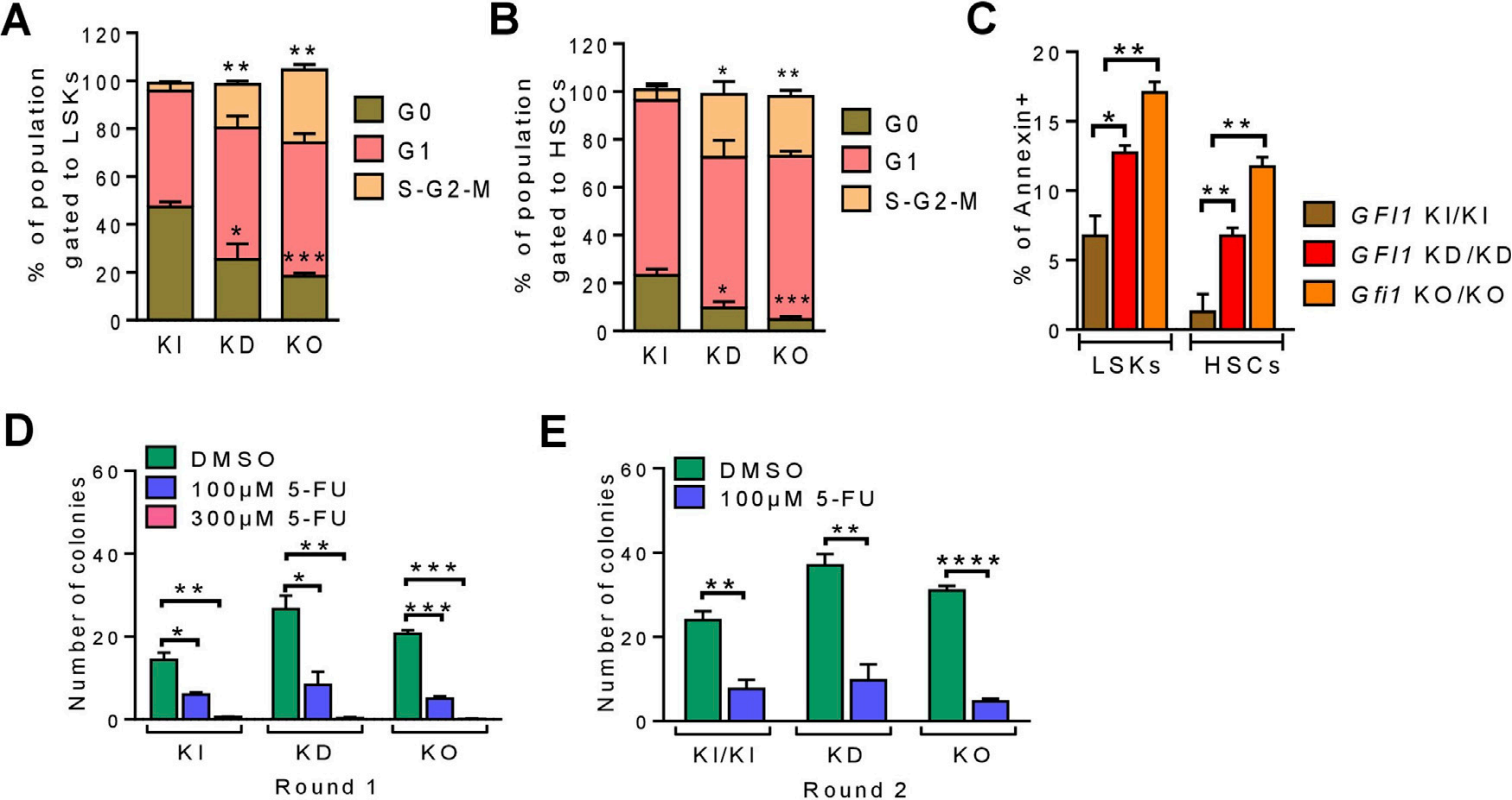
Reduced or loss of *GFI1* expression resulted in enhanced cell proliferation and apoptosis. **(A, B)**. The cell cycle status of *GFI1-*KI, -KD and -KO mice was measured in LSKs and HSCs populations (n ≥ 3; Avg ± SEM; *p ≤ 0.0336; **p ≤ 0.0027; ***p ≤ 0.0008). **(C)**. The apoptosis rate of *GFI1-*WT, -KD and -KO mice was measured by the percentage of annexin-positive cells in LSKs and HSCs populations (n = 3; Avg± SEM; *p = 0.0176; **p ≤ 0.0093). **(D, E)**. The CFU assay was performed from the HSCs of *GFI1*- KI, -KD and -KO mice with different doses of 5-FU as indicated. Quantification of the colonies represents the clonogenic potential of different HSCs populations in different conditions and rounds of plating [first round **(D)** and second round **(E)**] (n = 3; Avg ± SEM; *p ≤ 0.0151; **p ≤ 0.0069; ***p ≤ 0.0004; ****p ≤ 0.0001).

Next, we evaluated the proliferation status of LSKs, HSCs and progenitor cells in *GFI1*-WT, -KD and -KO mice by measuring the cell cycle and the apoptosis rate. Reduced expression or loss of *GFI1* significantly enhanced proliferation as demonstrated by the percentage of cells in the proliferative phases of G1 and S-G2-M (Figures 2A, B; Supplementary Figures S3A–C). We then measured the rate of apoptosis by flow cytometry. The low level or loss of *GFI1* resulted in a significantly enhanced apoptosis rate compared to *GFI1*-WT in LSKs, HSCs and progenitor cell populations. (Figure 2C; Supplementary Figure S3D). We subsequently monitored the clonogenic capacity of different HSCs *via* colony-forming units (CFU) assay. Initial plating (round 1) of HSCs, followed by replating (round 2) of HSCs from *GFI1*-KD and *Gfi1*-KO mice resulted in a significantly higher colony number underscoring their proliferative nature of the cells compared to HSCs from *GFI1*- KI mice (Figures 2D, E). The addition of the standard chemotherapeutic agent such as 5-Fluorouracil (5-FU) demonstrated that the *GFI1*-KD and *Gfi1*-KO HSCs were more sensitive to 5-FU treatment compared to *GFI1*-KI cells (Figures 2D, E).

**FIGURE 3:**
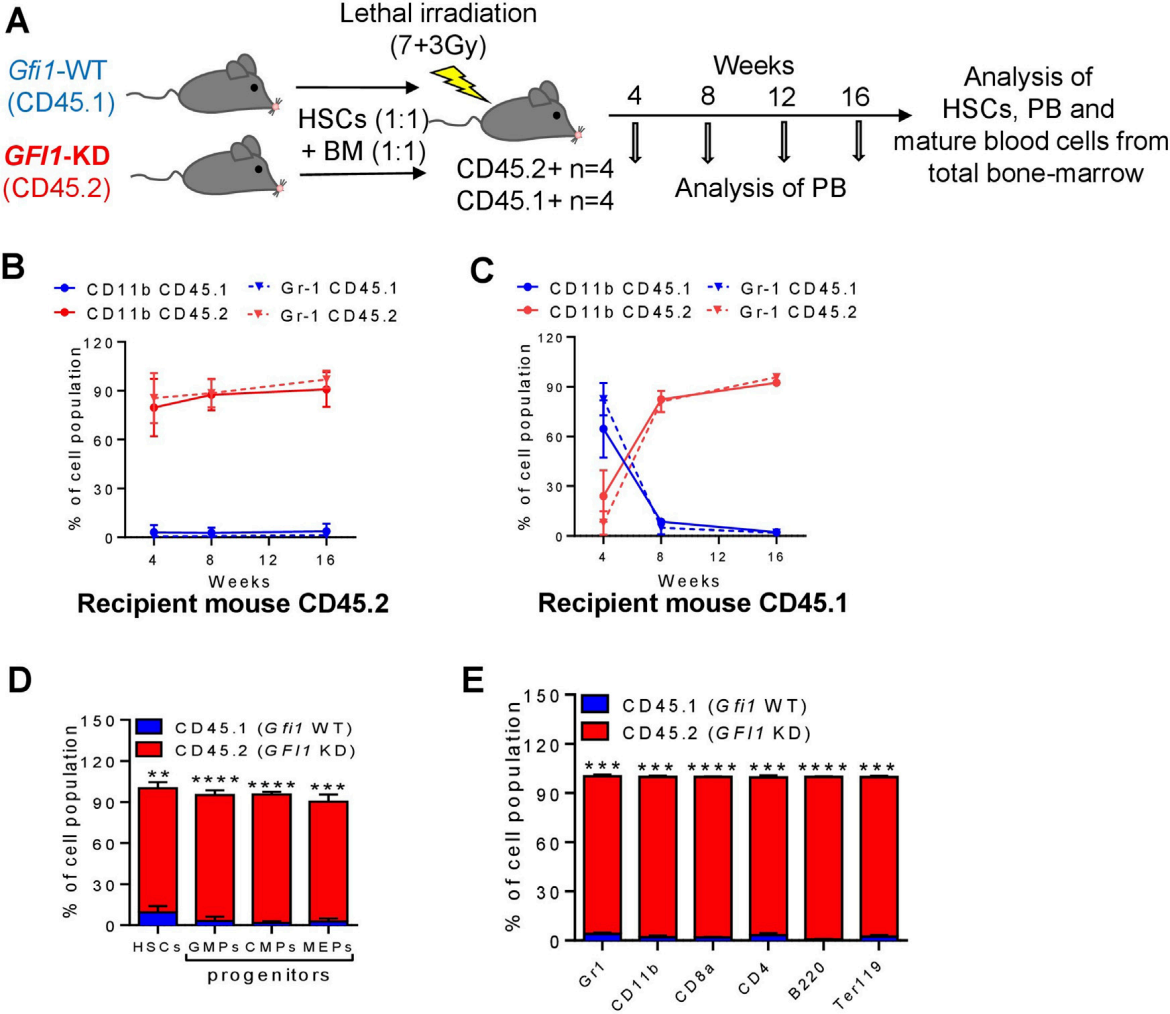
Mature cells were derived from CD45.2+ *GFI1*-KD HSCs in a competitive transplantation setting with CD45.1+ *Gfi1*-WT HSCs. **(A)**. Schematic representation of the competitive transplantation assay setup. HSCs were isolated from CD45.1 expressed *Gfi1*-WT mice and CD45.2-expressing *GFI1*-KD mice and were mixed in equal numbers (200 HSCs from each mouse) and transplanted into lethally irradiated C57Bl/6 recipient mice using 500,000 total BM cells as carrier cells (250,000 from each mouse). After the transplantation, the PB was analyzed at different time points and the BM was analyzed upon euthanization for the presence of HSCs and mature blood cells. **(B, C)**. CD45.1 and CD45.2 expression on the cell surface of myeloid (granulocytes and monocytes) cells in the peripheral (PB) were measured by flow cytometry at 4, 8, 12, and 16 weeks after transplantation into CD45.2+ and CD45.1 + recipient mice. Granulocytes were defined as Ly6G^hi^CD11b^+^, Monocytes were defined as Ly6G^int^CD11b^+^. **(D)** The percentage of HSCs and progenitor populations (GMPs, CMPs and MEPs) expressing CD45.1 and CD45.2 receptors, measured from the total BM cells (n = 3; Avg ± SEM; **p = 0.0061; ****p ≤ 0.0001). **(E)** The percentage of differentiated cell populations (gr1-granulocytes, CD11b-monocytes, CD8a-cytotoxic T cells, CD4^−^ T-Helper cells, B220-B lymphocytes, Ter119-erythrocytes) expressing CD45.1 and CD45.2 receptors, measured from the BM cells (n = 3; Avg ± SEM; ***p ≤ 0.0003; ****p ≤ 0.0001).

**FIGURE 4:**
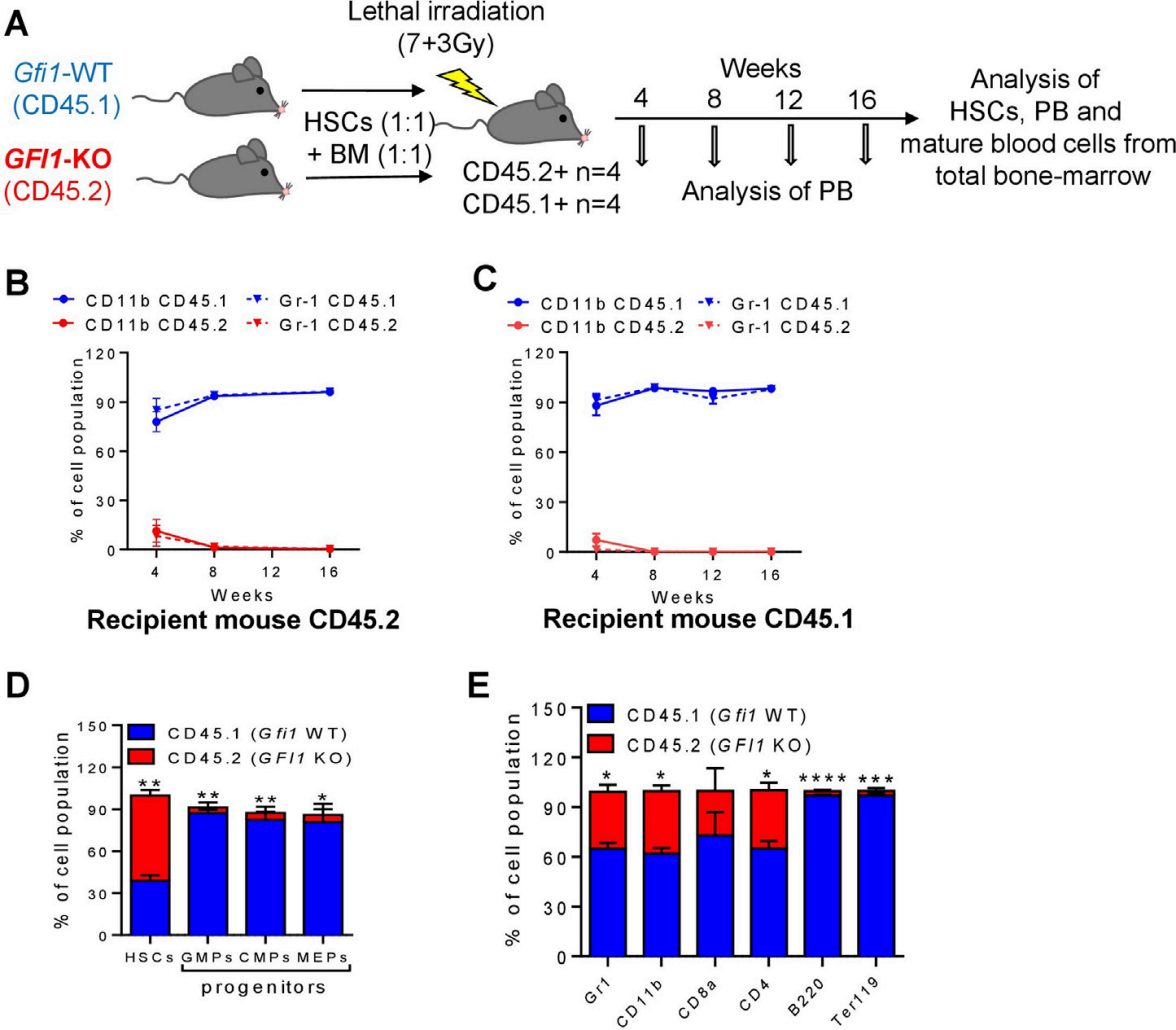
Mature cells were derived from CD45.1+ *Gfi1*-WT HSCs in a competitive transplantation setting with CD45.2+ *Gfi1*-KO HSCs. **(A)**. Schematic diagram of the competitive transplantation assay setup. HSCs were isolated from CD45.1 expressed *Gfi1*-WT mice and CD45.2-expressing *GFI1*-KO mice and were mixed in equal numbers (200 HSCs from each mouse) and transplanted into lethally irradiated C57Bl/6 recipient mice using 500,000 total bone-marrow cells as carrier cells (250,000 from each mouse). After the transplantation, the PB was analyzed at different time points and the BM was analyzed upon euthanization for the presence of HSCs and mature blood cells. **(B, C)**. CD45.1 and CD45.2 expression on the cell surface of myeloid (granulocytes and monocytes) cells in the PB were measured by flow cytometry at 4, 8, 12 and 16 weeks after transplantation into CD45.2+ and CD45.1 + recipient mice. Granulocytes were defined as Ly6G^hi^CD11b^+^, Monocytes were defined as Ly6G^int^CD11b^+^. **(D)** The percentage of HSCs and progenitor populations (GMPs, CMPs and MEPs) expressing CD45.1 and CD45.2 receptors was measured from the total BM cells (n = 3; Avg± SEM; *p = 0.0313; **p ≤ 0.0087). **(E)**. The percentage of differentiated cell populations (gr1-granulocytes, CD11b-monocytes, CD8a-cytotoxic T cells, CD4^−^ T-Helper cells, B220-B lymphocytes, Ter119-erythrocytes) expressing CD45.1 and CD45.2 receptors were measured from the total BM cells (n = 3; Avg ± SEM; *p ≤ 0.0433; ***p = 0.0005).

Next, we investigated how reduced levels or loss of *GFI1* affected the ability of HSCs to engraft post-transplantation and measured their competence to differentiate into the various cell lineages. To this end, we performed competitive transplantation assays as illustrated (Figure 3A; Figure 4A). We isolated HSCs and BM cells from CD45.1-expressing *Gfi1*-WT and CD45.2-expressing *GFI1*-KD or -KO mice and mixed 200 HSCs and 500,000 BM from each of the CD45.1+ and CD45.2 + mice and transplanted into lethally irradiated CD45.2 + C57BL/6 or CD45.1 + Gfi1-WT recipient mice. Usage of both CD45.1+ and CD45.2 + recipient mice ruled out a possible background bias. Post transplantation, every 4 weeks we analysed the PB for CD45.1 and CD45.2 expression on different myeloid cells (monocytes- CD11b+ and granulocytes-Gr1+), T cells (CD8^+^ and CD4^+^), B cells (B220+) and erythroid cells (Ter119+). After 16 weeks of transplantation, we euthanized the mice and analyzed them for mature and progenitor cells and HSCs of the PB and BM. We found that in the competitive setting involving *GFI1*-KD mice, the number of CD45.2 + mature cells analysed in the PB increased over time and nearly reached 100% in 16 weeks after transplantation (Figures 3B, C; Supplementary Figures S4A, B). The presence of short-lived myeloid cells derived from CD45.2 + *GFI1*-KD cells 4 weeks after transplantation into CD45.2 + host mice rules out a potential carry-over of these cells as a result of the transplantation (Figure 3B). 8 weeks after transplantation, about 90% of all monocytes in PB were CD45.2+ in both transplantation settings, hence originating from CD45.2 + *GFI1*-KD HSCs in the competitive transplantation assay (Figures 3B, C). Erythroid cells, B-cells and long-lived cells like T-cells are derived from CD45.2 + *GFI1*-KD HSCs and reached 90% after 16 weeks in both the CD45.2 and CD45.1 recipient mice (Supplementary Figures S4A, B). It has previously been shown that long-term reconstitution can be determined 12–16 weeks after transplantation (Morrison and Weissman, 1994; Akala et al., 2008). Hence, to determine the reconstitution capacity in the competitive setting, the mice were sacrificed after 16 weeks of transplantation and measured the percentage of HSCs, progenitors (CMPs, GMPs and MEPs) and differentiated cells from the total BM. The BM analysis showed that the major proportion of HSCs, progenitors and differentiated cells were derived from CD45.2 cells (Figures 3D, E). This indicates *GFI1*-KD HSCs possess reconstitution capacity, indeed long-term HSC activity and that GFI1 has a dose-dependent effect in contribution to PB cell formation when transplanted in a competitive setting with *Gfi1*-WT HSC.

In contrast to the results from the *GFI1*-KD transplantations, CD45.2-derived cells from *Gfi1*-KO mice were not present in the *Gfi1*-KO competitive transplantation setting (Figures 4A–E; Supplementary Figures S4A, B). About 4 weeks after transplantation the percentage of CD45.2-derived monocytes and granulocytes was less than 11% in both the CD45.2 and CD45.1 recipient mice (Figures 4B, C). Moreover, T cells, B-cells and erythroid cells were contributed from CD45.1-expressing HSCs even 16 weeks after transplantation (Supplementary Figures S5A, B). This finding is in line with previous publications that *Gfi1*-KO cells do not contribute to mature cells (Hock et al., 2004; Zeng et al., 2004). However, analysis of total BM revealed that the majority of HSCs are CD45.2, whereas the progenitor populations and differentiated cells were derived from CD45.1 HSCs (Figures 4D, E).

To measure self-renewal and clonogenic capacity *in-vivo* in a competitive setup, secondary transplantation was performed after euthanizing and analysing the BM from the primary transplanted mice. 10 Mio total BM cells from the primary transplanted mice (*GFI1*-WT/KD and *GFI1*-WT/KO groups) were collected and transplanted into lethally irradiated CD45.1 recipient mice (Supplementary Figure S6A). The PB was analysed for the expression of CD45.1 and CD45.2 on mature cells. After 8 weeks, mice transplanted with *GFI1*-WT/KD cells expressed CD45.2 receptors, hence the majority of the blood cells were derived from *GFI1*-KD mice (Supplementary Figure S6B), similar to the results from the primary transplanted mice. On the other hand, the mature cells from the mice transplanted with *GFI1*-WT/KO group expressed CD45.1 receptors, indicating their origin from *GFI1*-WT mice (Supplementary Figure S6C).

**FIGURE 5:**
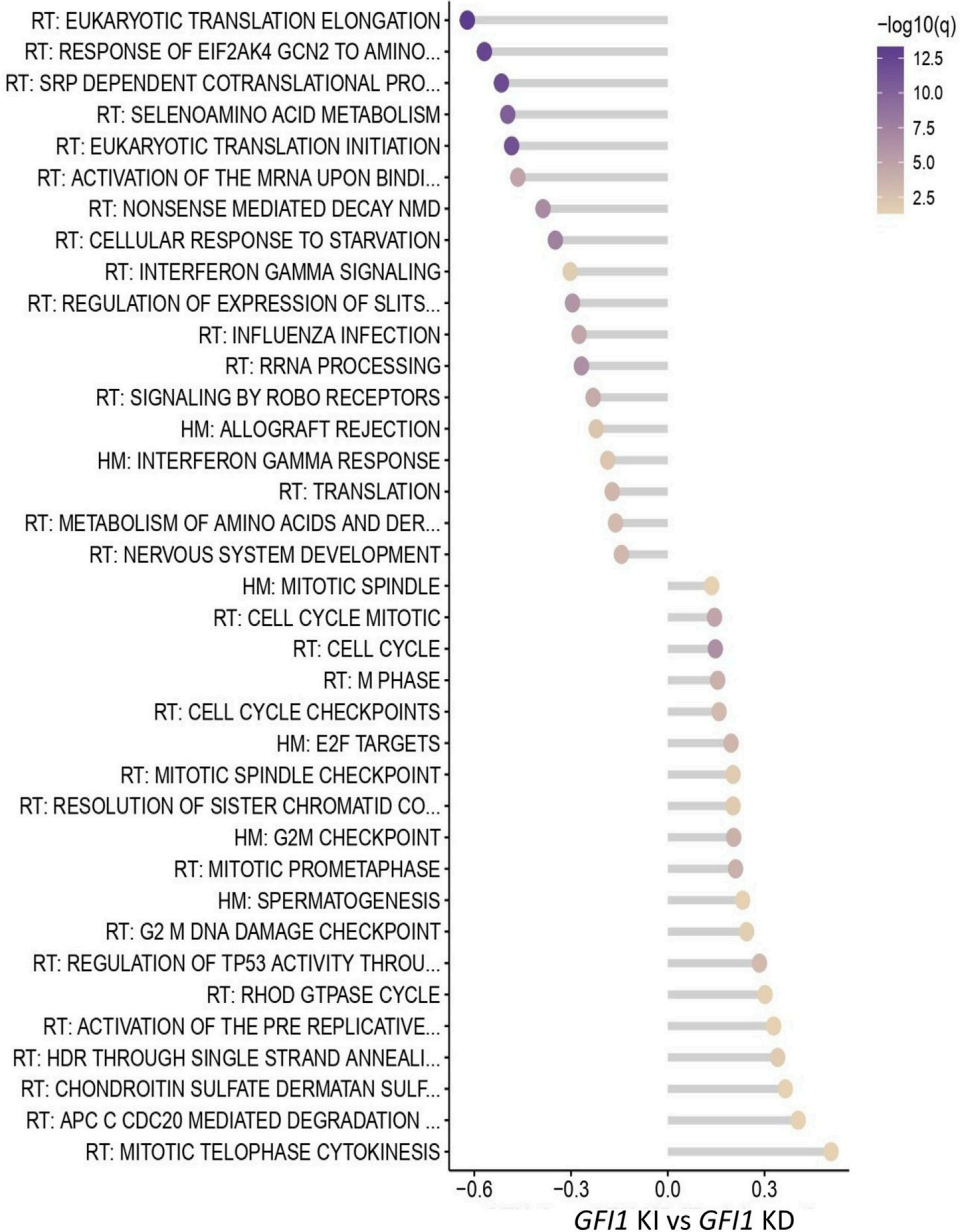
RNA-seq data indicate differential regulation of cell-cycle-related pathways in *GFI1*-KD HSCs. Pathway analysis from the RNA-seq data shows significantly altered pathways in *GFI1*-KD HSCs in comparison with *GFI1*-KI HSCs.

To investigate the low and loss of *GFI1* induced molecular and global gene expression changes, we performed RNA-seq analysis in HSCs derived from *GFI1*-KD or *Gfi1*-KO mice and compared it to *GFI1*-KI mice. The RNA-seq data analysis demonstrated there are indeed distinct differences in GFI1-KD/-KO/-KI mice. Genes involving significant alterations in expressions are depicted in Supplementary Figures S7A, B. Further analysis revealed that 89 and 95 genes are differentially expressed in *GFI1*-KI vs. -KD and *Gfi1*-WT vs. KO settings, respectively. To better illustrate the differences at the pathway levels, the gene set enrichment analysis (GSEA) was performed against the combined Reactome (RT) and hallmark (HM) genesets and the significantly altered pathways in *GFI1*-KD and *Gfi1*-KO in comparison with *GFI1*-KI mice and *Gfi1*-WT mice respectively was identified. Pathway analysis identified that the cell cycle and related pathways such as G2M checkpoints, and DNA damage checkpoints were significantly upregulated in *GFI1*-KD HSCs (Figure 5). On the contrary, these pathways were downregulated in *Gfi1*-KO HSCs (Supplementary Figure S8). Interestingly it was also previously shown that GFI1 plays a role in overriding the G1 cell-cycle checkpoint in T-cells and contributing to T-cell lymphomagenesis (Karsunky et al., 2002b). Our observations as cell cycle regulation, thus shed light on different molecular alterations induced by varied *GFI1* expression.

## 4 Discussion

GFI1 influences the prognosis of AML, CML and MPN patients in a dose-dependent manner, and reduced levels of *GFI1* accelerated disease development in murine models of human leukemia (Kok et al., 2013; Hönes et al., 2016; Volpe et al., 2017; Vadnais et al., 2018). AML, CML and MPN arise as a result of the transformation of HSCs or early hematopoietic progenitors that still possess some degree of stem cell capacity (Goardon et al., 2011). It has been described that complete loss of Gfi1 leads to an impaired function of HSCs (Hock et al., 2003; Zeng et al., 2004) and an increase in LSK frequency, possibly due to increased proliferation (Hock et al., 2003; Khandanpour et al., 2011). It has also been previously postulated that *Gfi1*-KO cells fail to compete with *Gfi1*-WT cells in a competitive setting, demonstrated by a lack of reconstitution capacity of the myeloid and lymphoid compartments in the PB (Zeng et al., 2004). The question arising from these results is whether the lack of mature cells in a competitive transplantation setting is a) due to a failure of *Gfi1*-KO HSCs to engraft, b) due to a lack of survival of *Gfi1*-KO HSCs in competition with *Gfi1*-WT HSCs or c) due to a loss of *Gfi1*-KO HSC capacity to contribute to PB cell production. We were hence interested to determine whether reduced levels of GFI1 or loss of Gfi1 influence a) the number of HSCs and b) their ability to engraft and reconstitute the PB and BM of recipient mice in a competitive transplantation setting.

We expand our knowledge about the distinct role of GFI1 in various cellular populations by showing that reduced levels of *GFI1* and loss of *Gfi1* lead to the significant expansion of LSK cells, but to a highly significant reduction of HSCs. On a functional level, reduced expression or loss of *GFI1* expression did not dramatically impede the ability of *GFI1*-KD or *Gfi1*-KO HSCs to engraft or to expand after transplantation (up to 80% of HSCs were of *GFI1*-KD origin and up to 60% of HSCs were of *Gfi1*-KO origin). Yet, loss of *GFI1* negatively influenced the maturation of HSCs into the lymphoid and myeloid lineages when Gfi1 was absent but enhanced the ability to give rise to progenitor cells and mature cells if *GFI1* level were reduced. Lymphoid cells are generally long-lived, if they were generated from the transplanted HSCs early after transplantation, these cells would still be detectable at later time points, even though HSCs do not self-renew and differentiate anymore. Our data are in line with other reports highlighting the role of Gfi1 in lineage choice, similar to that of other transcription factors such as IRF8 and PU.1 (Spooner et al., 2009; Olsson et al., 2016). We thus hypothesized that knockdown and loss of GFI1 might regulate the choice of symmetric and asymmetric division of HSCs which could explain the leukemia-propagating function of reduced GFI1 levels in CML and AML (Hönes et al., 2016). The differential effect on the self-renewal and differentiation status in the *in-vivo* environment is further evidenced by upregulated cell-cycle related pathways in *GFI1*-KD HSCs while the opposite effect in *Gfi1*-KO HSCs.

However, further work is required to dissect this and get a deeper insight into which of the different epigenetic and gene-expression changes are induced by different *GFI1* levels and how this influences the function of HSCs. Nevertheless, our work lays the foundation to dissect this in more detail in future studies.

In conclusion, HSCs with reduced expression of GFI1 could self-renewal and reconstitute, but HSCs with a complete loss of GFI1 only have the self-renew but lose reconstitution ability.

## Data Availability

The original contributions presented in the study are included in the article/Supplementary Material, further inquiries can be directed to the corresponding author.

## Supplementary material

SUPPLEMENTARY FIGURE S1 Schematic representation of the gating strategy of LSKs, HSCs and progenitor populations for FACS analysis. The total bonemarrow cells were gated for the live cell population, followed by selecting the lineage-depleted population and further gating to Sca1+, c-Kit+ (L-S+K+), and Sca1-, c-Kit+ (L-S-K+). The L-S+K+ population is further gated to CD150+ and CD48- for HSCs. While, the L-S-K+ population is gated to CD34 and CD16/32 for CMPs, GMPs, and MEPs.

SUPPLEMENTARY FIGURE S2 HSCs, LSKs and progenitor cells distribution in GFI1-KD and Gfi1-KO mice compared to GFI1-WT mice. **(A, B)**. Reduced levels of GFI1 (GFI1-KD) and loss of Gfi1 (Gfi1-KO) led to an increase in the percentage of LSK (Lin-Sca1+c-Kit+) and HSCs compared to GFI1 WT (+/+, +/KI, KI/KI) in the total BM cells (n ≥ 3; Avg ± SEM; **p* = 0.0465; ***p* ≤ 0.0066). **(C, D)**. The change in the percentage of LSKs and HSCs is not affected by age. The GFI1-KD and GFI1-KO mice have more LSKs and fewer HSCs percentages compared to GFI1-WT in both the young and old mice. (n = 3; Avg ± SEM; **p* ≤ 0.0493; ***p* = 0.0017). **(E, F)**. GFI1-KD and Gfi1-KO mice have more GMPs compared to GFI1-WT mice, while no change in the level of CMPs and MEPs (n ≥ 3; Avg ± SEM; **p* = 0.0113; ***p* ≤ 0.0039).

SUPPLEMENTARY FIGURE S3 The cell cycle status and apoptosis rate in different progenitor fractions in GFI1-KD and Gfi1-KO mice compared to GFI1-WT mice. **(A-C)**. Reduced levels of GFI1 (GFI1-KD) and loss of Gfi1 (Gfi1-KO) led to a significant reduction in the number of cells in the G0 phase while an increase in the G1 phase in CMPs, GMPs, and MEPs fractions of whole bonemarrow analysis (n ≥ 3; Avg ± SEM; **p* ≤ 0.0427; ***p* = 0.0050). **(D)**. Low level (GFI1-KD) or loss of GFI1 (Gfi1-KO) leads to an increased rate of apoptosis (Annexin positive) in CMPs, GMPs and MEPs fraction compared to GFI1-KI mice (n = 3; Avg ± SEM; **p* = 0.0176; ** = 0.0020).

SUPPLEMENTARY FIGURE S4 GFI1-KD HSCs contribute to progenitor cell development in the competitive transplantation assay with Gfi1-WT HSCs. Mature cells such as cytotoxic T cells (CD8+), T-helper cells (CD4+), erythroid lineage (Ter119+), B-cells (B220+) and the total BM cells were derived from GFI1-KD HSCs expressing CD45.2 receptor upon co-transplanting with Gfi1-WT HSCs expressing CD45.1 receptors into both CD45.2 **(A)** and CD45.1 **(B)** recipient mice. The Y-axis represents the percentage of the particular cell population expressing the receptors CD45.1 or CD45.2.

SUPPLEMENTARY FIGURE S5 Gfi1-WT HSCs contribute to progenitor cell development in the competitive transplantation assay with GFI1-KO HSCs. Mature cells such as cytotoxic T cells (CD8+), T-helper cells (CD4+), erythroid lineage (Ter119+), B-cells (B220+) and the total BM cells were derived from Gfi1-WT HSCs expressing CD45.1 receptor upon co-transplanting with Gfi1-KO HSCs expressing CD45.2 receptors into both CD45.2 **(A)** and CD45.1 **(B)** recipient mice. The Y-axis represents the percentage of the particular cell population expressing the receptors CD45.1 or CD45.2 in the peripheral blood.

SUPPLEMENTARY FIGURE S6 Peripheral blood analysis of secondary transplantation. **(A)**. Schematic presentation of the secondary transplantation of the total bone marrow cells from the primary co-transplanted mice into lethally irradiated CD45.1 recipient mice, followed by peripheral blood analysis at 4 and 8 weeks after transplantation. **(B)**. Peripheral blood analysis displays, BM cells from the GFI1-KD (CD45.2) mice contribute to mature blood cells after 4 and 8 weeks of secondary transplantation in GFI1 WT/KD setup (n = 3; Avg ± SEM; ***p* ≤ 0.0083; ***≤0.0005; ****≤ 0.0001). **(C)**. Peripheral blood analysis displays, BM cells from the Gfi1-WT (CD45.1) mice contribute to mature blood cells after 4 and 8 weeks of secondary transplantation in GFI1 WT/KO setup (n = 3; Avg ± SEM; **p* = 0.0196; **≤0.0083; ****≤0.0001).

SUPPLEMENTARY FIGURE S7 The volcano plots indicating the gene expression signature measured by RNA-seq analysis. RNA-seq analysis from the HSCs of GFI1-KI, Gfi1-WT, GFI1-KD and Gfi1-KO mice indicates the differentially expressed genes in GFI1-KD **(A)** and Gfi1-KO **(B)** mice compared to GFI1-KI and Gfi1-WT mice, respectively.

SUPPLEMENTARY FIGURE S8 RNA-seq data indicate differential regulation of cell-cycle-related pathways in Gfi1-KO HSCs. Pathway analysis from the RNA-seq data shows significantly altered pathways in Gfi1-KO HSCs in comparison with Gfi1-WT HSCs.
