# Supplementary figures and images for: Dose-dependent effect of GFI1 expression in the reconstitution and the differentiation capacity of HSCs
Source: Front Cell Dev Biol. 2023 Apr 5;11:866847. doi: 10.3389/fcell.2023.866847 (PMC10113925; doi:10.3389/fcell.2023.866847)

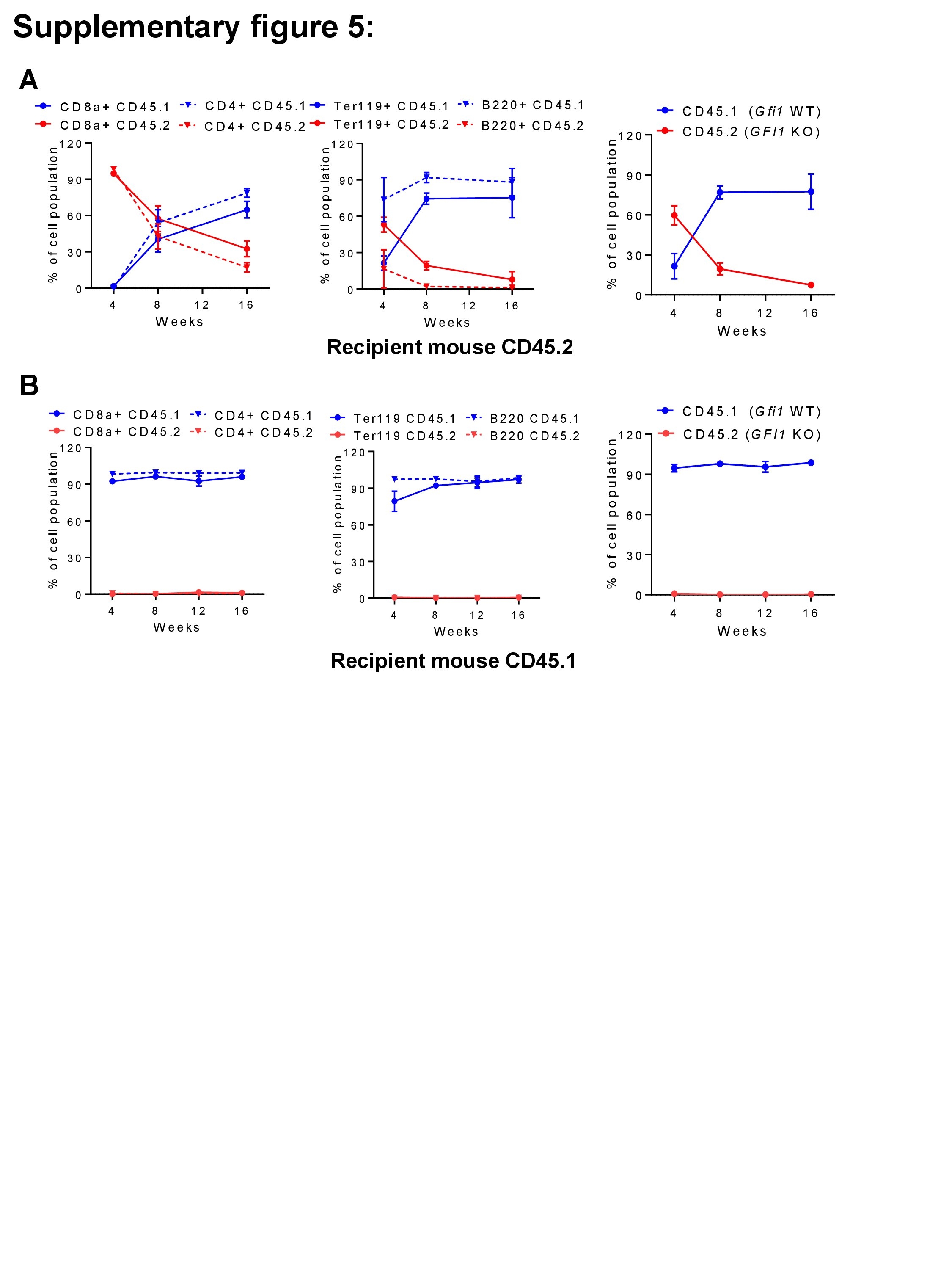

Supplement: Supplementary file 1 [file Image5.jpg]

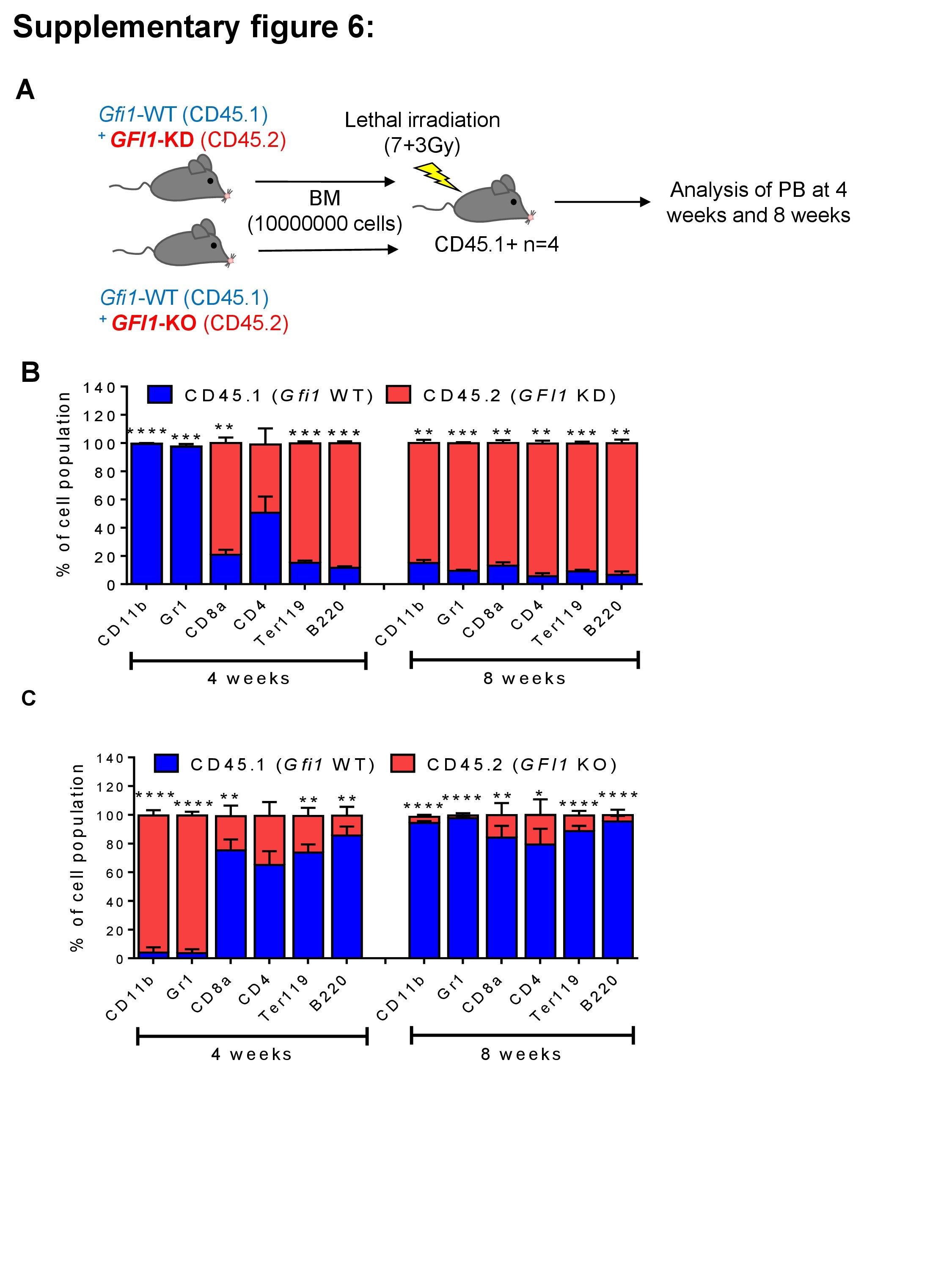

Supplement: Supplementary file 2 [file Image6.jpg]

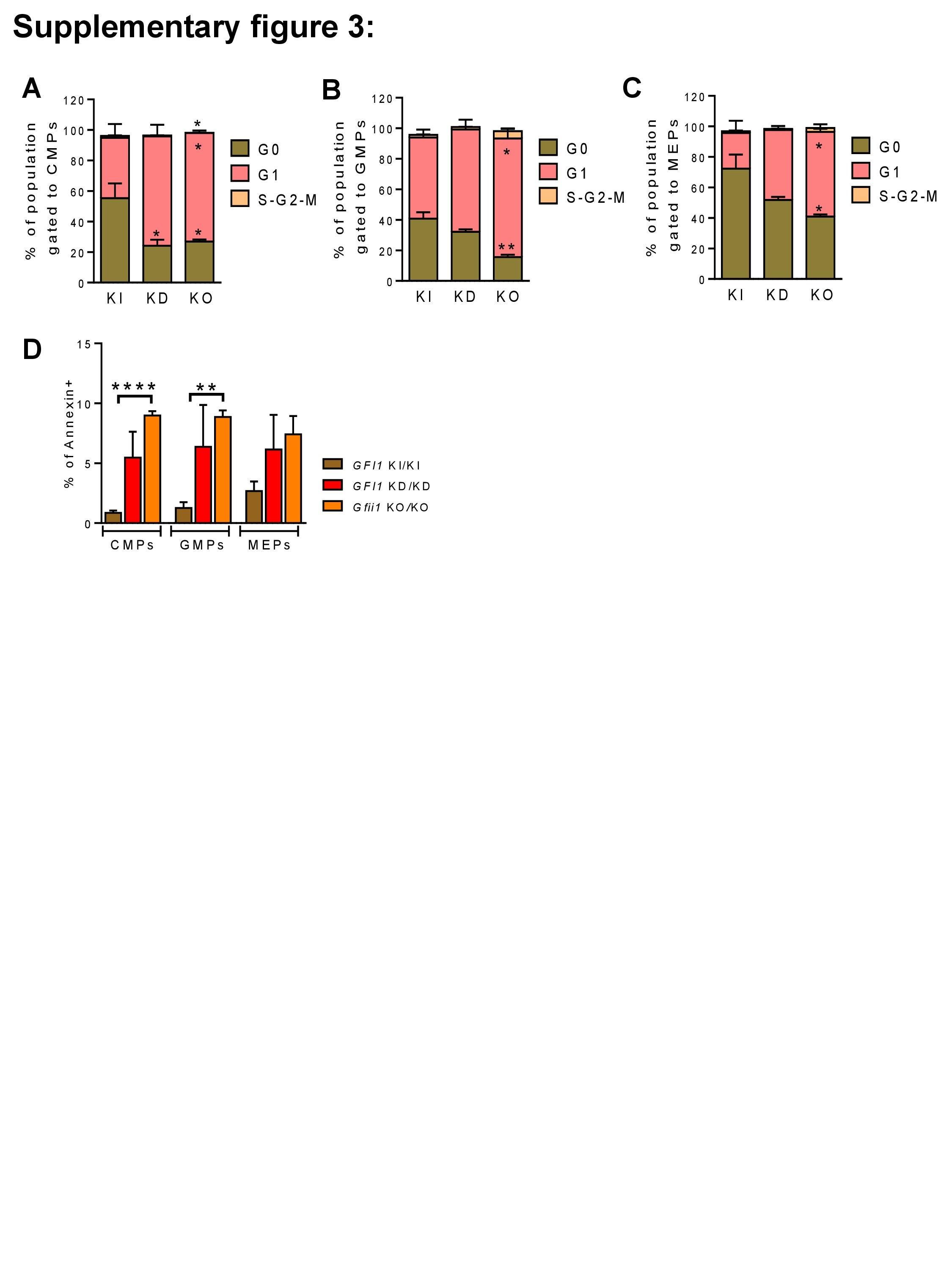

Supplement: Supplementary file 3 [file Image3.jpg]

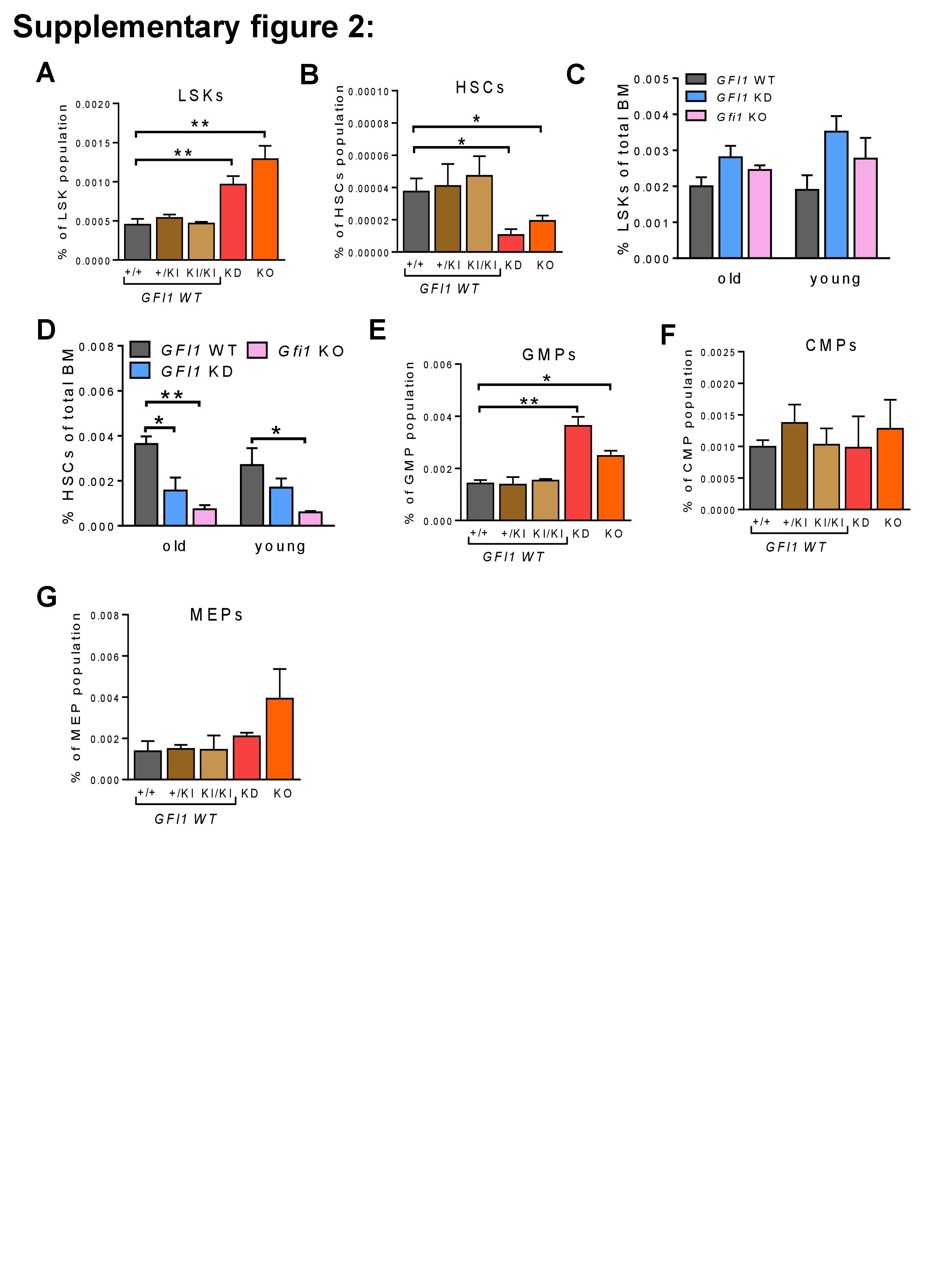

Supplement: Supplementary file 4 [file Image2.jpg]

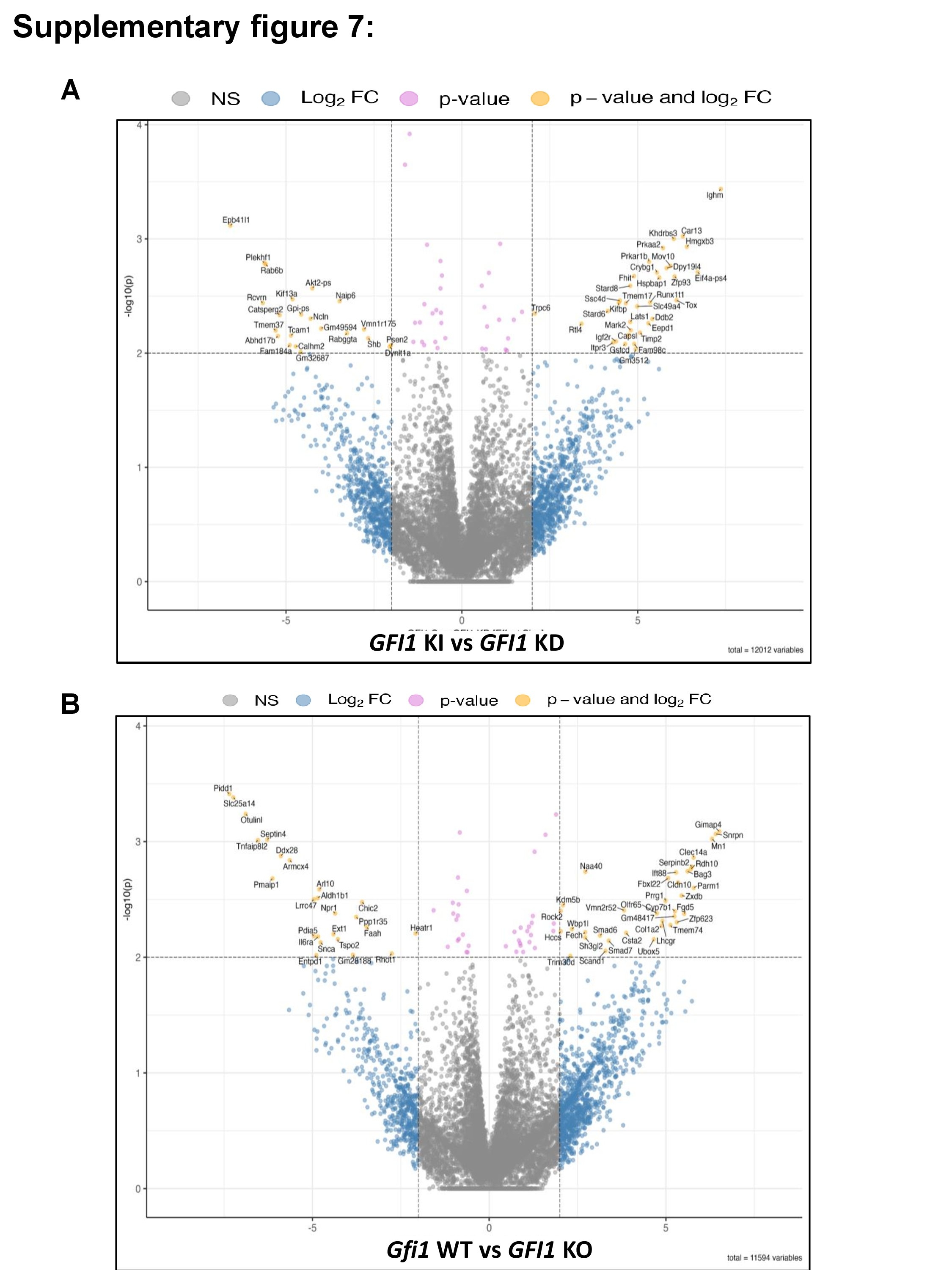

Supplement: Supplementary file 6 [file Image7.jpg]

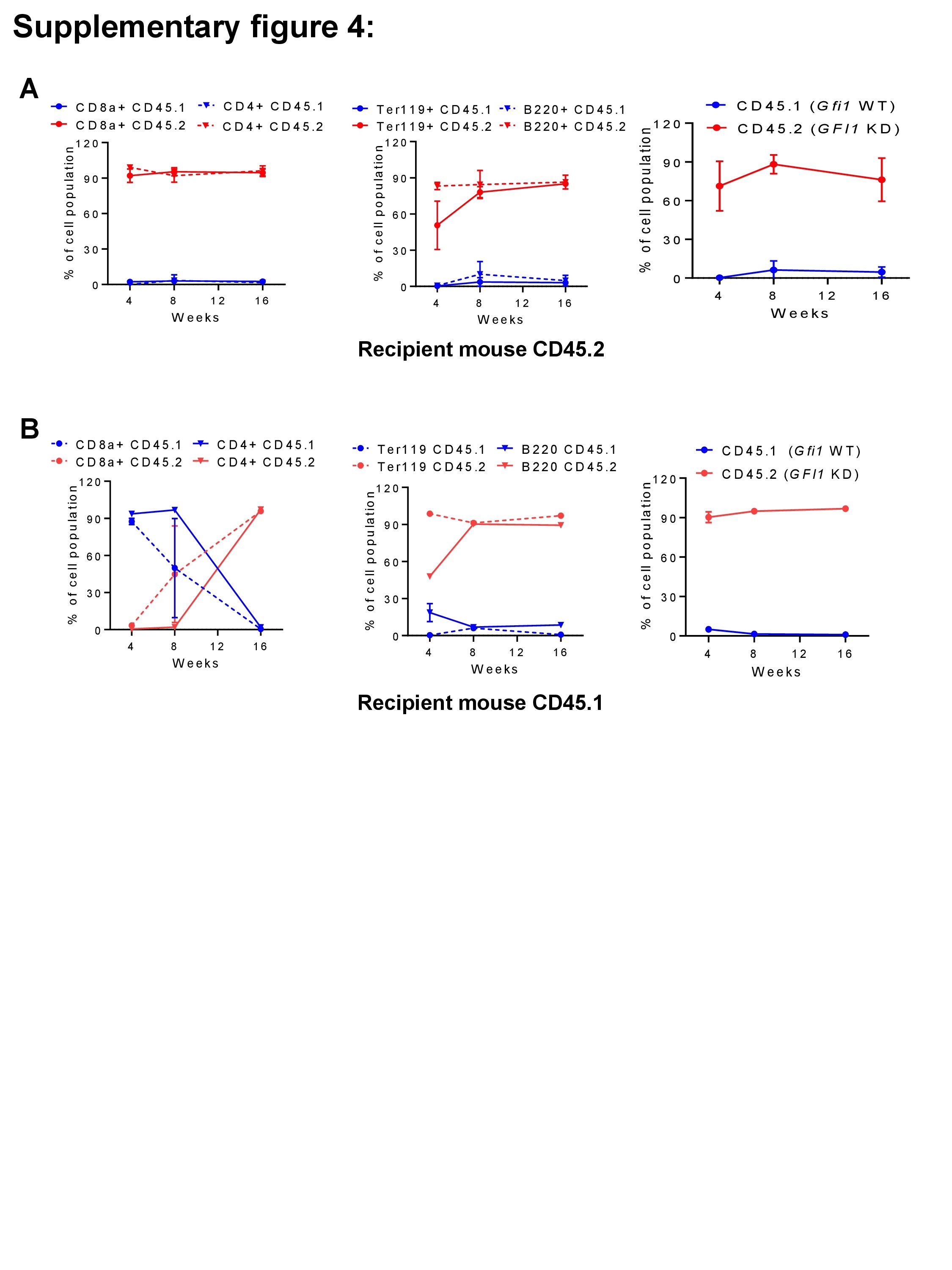

Supplement: Supplementary file 7 [file Image4.jpg]

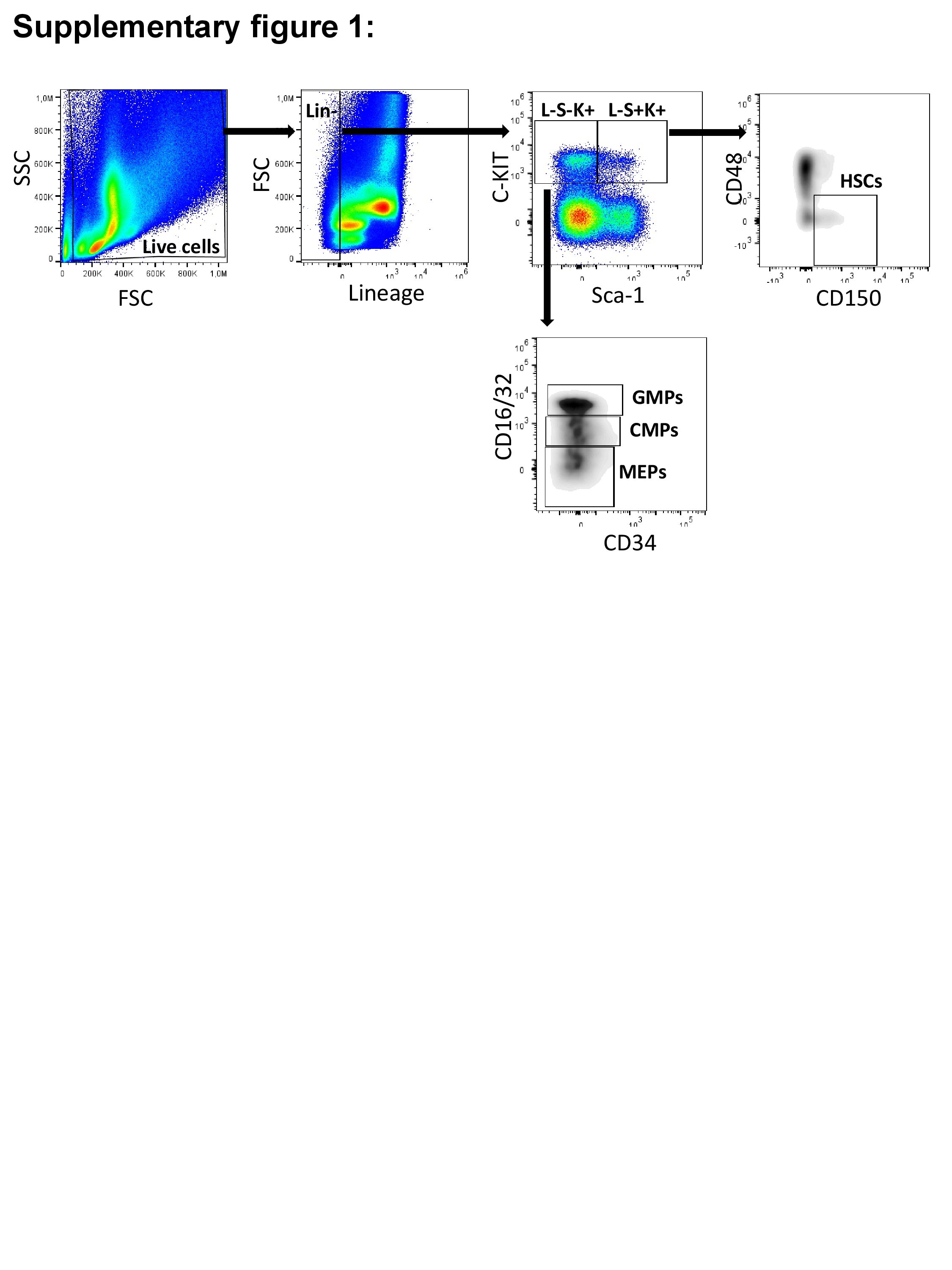

Supplement: Supplementary file 8 [file Image1.jpg]

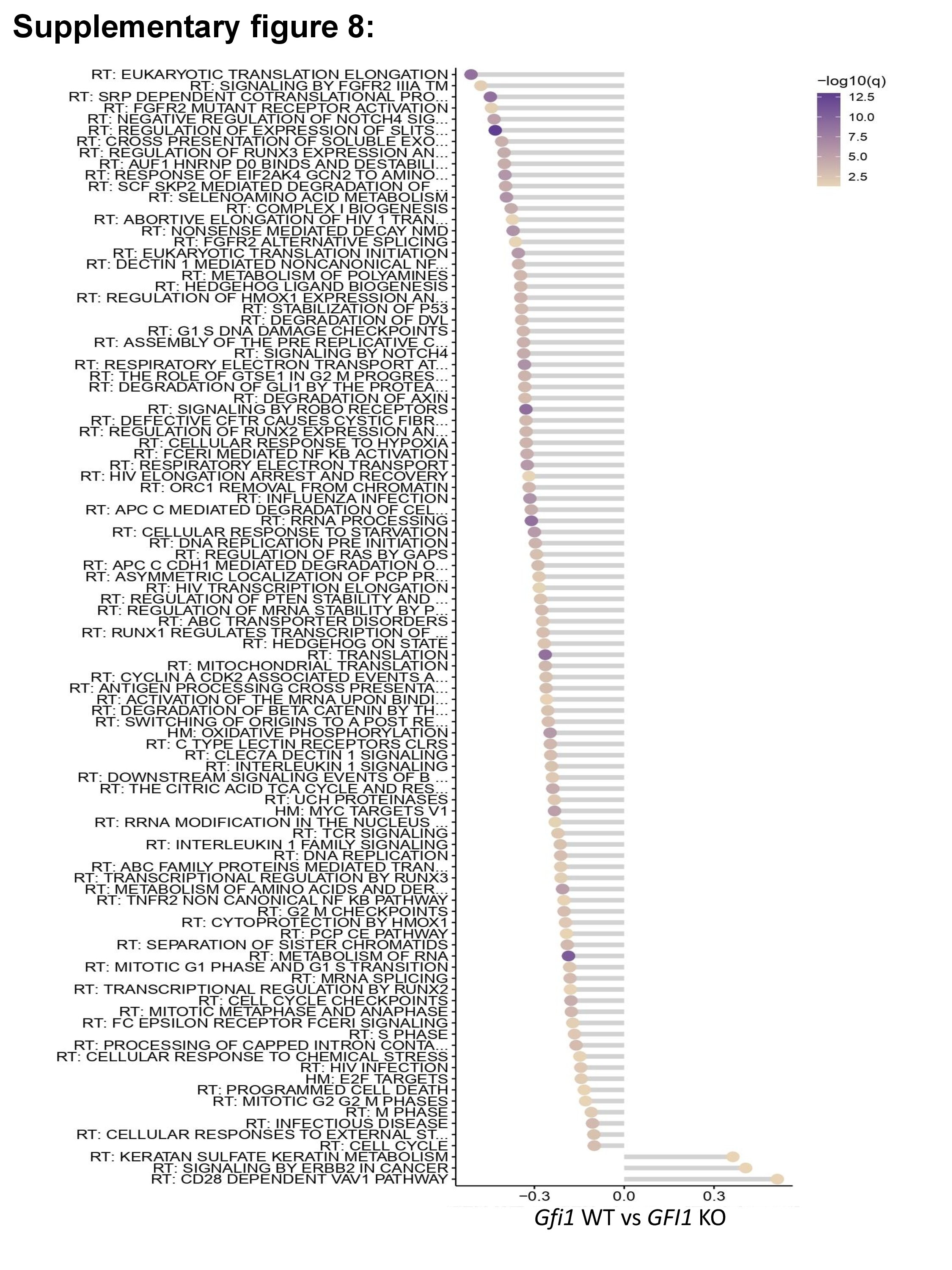

Supplement: Supplementary file 9 [file Image8.jpg]
