# Supplementary material for: Dose-dependent effect of GFI1 expression in the reconstitution and the differentiation capacity of HSCs
Source: Front Cell Dev Biol. 2023 Apr 5;11:866847. doi: 10.3389/fcell.2023.866847 (PMC10113925; doi:10.3389/fcell.2023.866847)
Supplement: Supplementary file 5 [file Table1.DOCX]

Supplementary Table 1:

| **Peripheral blood and bone marrow analysis** | | | |
| --- | --- | --- | --- |
| **Staining For** | **Surface Marker** | **Fluorochrome** | **Catalog No** |
| Granulocytes | Gr-1 | FITC | 108405 (Biolegend) |
| Monocytes | CD11b | APC | 101211 (Biolegend) |
| Erythrocytes | Ter119 | APC | 116211 (Biolegend) |
| B Cells | B220 | FITC | 103205 (Biolegend) |
| Cytotoxic T-cells | CD8a | APC | 100711 (Biolegend) |
| Helper T-cells | CD4 | FITC | 100405 (Biolegend) |
| CD45.1 | CD45.1 | PerCP-Cy5.5 | 110727 (Biolegend) |
| CD45.1 | CD45.2 | PE | 109808 (Biolegend) |
|  | | | |
| **LSKs and HSCs** | | | |
| Lineage negative-Streptavidin | | BV605 | 405229 (Biolegend) |
| Sca1 | | APC-Cy7 | 108125 (Biolegend) |
| C-Kit | | BV421 | 105827 (Biolegend) |
| CD150 (SLAM) | | PE | 115904 (Biolegend) |
| CD48 | | APC | 103411 (Biolegend) |
| CD45.1 | | PerCP-Cy5.5 | 110727 (Biolegend) |
| CD45.2 | | FITC | 109805 (Biolegend) |
|  | | | |
| **CMPs, GMPs and MEPs** | | | |
| Lineage negative-Streptavidin | | BV605 | 405229 (Biolegend) |
| Sca1 | | APC-Cy7 | 108125 (Biolegend) |
| C-Kit | | BV421 | 105827 (Biolegend) |
| CD16/32 | | PE | 156606 (Biolegend) |
| CD34 | | APC | 128612 (Biolegend) |
| CD45.1 | | PerCP-Cy5.5 | 110727 (Biolegend) |
| CD45.2 | | FITC | 109805 (Biolegend) |
